# A Fetal Hemolytic Anemia in a Child with Cytomegalovirus Infection

**Published:** 2014-04-20

**Authors:** S Hosseeini, Sh Ansari, E Kalantar, M Sabzechian, A Alibeik, A Dorgalaleh

**Affiliations:** 1 Aliasghar Hospital, Iran University of Medical Sciences, Tehran, Iran; 2 Gholhak Laboratory, Shariaty Street, Tehran, Iran; 3 Immunology Department, Iran University of Medical Sciences, Tehran, Iran; 4 hematology and blood transfusion Department, Allied Medical School, Tehran Unversity of Medical Sciences, Tehran, Iran

**Keywords:** Autoimmune hemolytic anemia, Cytomegalovirus, Direct anti globulin test, Viral load

## Abstract

**Background:**

Autoimmune hemolytic anemia is a hematologic disorder that is rarely observed in infants and young children. Most of the cases are associated with viral or bacterial infections. In some cases, AIHA can be characterized by a chronic course and an unsatisfactory control of hemolysis, thus requiring prolonged immunosuppressive therapy.

**Case report:**

Especially in children younger than 2 years of age, the clinical course of the disease may show either resistance to steroids or dependence on high-dose steroids. We report here an infant fatal autoimmune

**Conclusion:**

This case suggests that investigation for the presence of CMV infection in infantile AIHA should be considered. Severe hemolysis is rare but could be a potentially life-threatening complication of CMV infection described mostly in immune compromised adults and children.

## Introduction

Autoimmune hemolytic anemia (AIHA) refers to a collection of disorders characterized by the presences of auto antibodies which bind to the patient's own erythrocytes, leading to premature red cell destruction. Specific characteristics of the auto antibodies, especially the type of antibody, optimal binding temperature (warm or cold) and whether complement is fixed or not influences the clinical picture of the disease. In all cases of AIHA, however, the presence of autoantibody leads to a shortened red blood cell survival and when the rate of hemolysis exceeds, the ability of bone marrow to replace the destroyed red cells, anemia develops (1-3).

AIHA is rarely observed in infants and young children. Most of the cases are associated with viral or bacterial infection, but the immunologic events leading to hemolysis are poorly understood (3-6). 

Cytomegalovirus (CMV) is a common viral agent responsible for a wide range of clinical manifestations that vary according to the immunologic status of the patient immune compromised adults and children. CMV infection can leads to severe clinical manifestations related to direct viral cytotoxic effect on specific organs and tissues (gastrointestinal tract, retina, and hematopoietic system). Hemolytic anemia due to acute CMV infection in an immune competent adult had been reported (7-9). 

Acute infections with CMV may lead to severe hematologic disorders. CMV infection can also be associated with other manifestations, including hemolytic anemia (10, 11).

Severe hemolysis is a rare but potentially life-threatening complication of CMV infection described mostly in immune compromised adults and children (5, 12).

We describe here a refractory warm Coombs-positive hemolytic anemia in a child with acute CMV infection not responding to different lines of therapy.

## Case report

On May 2010 a 4 month old boy was referred to Aliasghar hospital with acute autoimmune hemolytic anemia, transfusion dependent and not responding to prednisolone. At admission he had pallor, fever, icter, dark urine and on physical examination a splenomegaly (2cm) below the ribs was noticeable.

The patient was an offspring of a family marriage (cousins), with mother having a history of three abortions, one with a heart anomaly, one with external pregnancy (EP) and the last, with oligo hydroaminious. 

His mother's TORCH (toxoplasmosis, 'other', rubella, cytomegalovirus and herpes) study was as follows which according to this data was negative.

The laboratory assessment of patient at the time of admission revealed following results:

WBC: 8.1*103/μl, Hemoglobin: 5.8g/dl, Hematocrit: 17.5%, MCV: 87.6 fI, MCH: 26.6 pg,

Plt: 157*103/μl, Retic: 21%[increased, Neut: 70%, lymp: 27%, Baso: 1%, Mono: 2%, NRBC:

3/100, LDH: 3496U/L [increased], Alk: 226 U/L, SGOT: 136 U/L[increased], SGPT: 80

U/L[increased], G6PD: sufficient, cold agglutination: (Negative), Blood group: (O positive),

Direct Combs: positive (3+) with (anti -IgG, anti- C3d), Indirect combs: Positive (2+), and micro spherocyte (1+) and red blood cell agglutination (2+), polychromasia (2+) were the prominent morphologies in the peripheral blood smear (PBS).

Consequently EBV, CMV, and viral hepatitis antibodies were sent to clinical laboratory for more evaluation of case scenario behind the hemolytic events.

According to the laboratory data [anemia, reticulocytosis, and positive coombs test] AHIA was confirmed and an ABO and Rh compatible packed RBC was ordered and prednisolon econtinued and he also received high dose of methyl prednisolone (HDMP) (30mg/kg/day), IVIG [40mg/kg/4day/week), antibiotic and folic acid (1mg/day).Since cross match compatible blood was not available in the hospital blood bank. Patient's blood was sent to Iranian blood transfusion organization (IBTO) for antibody detection and identification and due to the presence of warm auto-Anti-e and auto anti-C, a group compatible C-Ag and e - Ag negative packed RBC was prepared and transfused. Consequently patient was discharged from hospital with a stable condition.

He was again admitted to the hospital on June 2010 with fever, productive cough and icter and the lab results evaluated at that time were as follows WBC: 14.5*103/μl, RBC: 2.36mil/ul, Hemoglobin: 7.7g/dl, Hematocrit: 23.6%, MCH: 32.4pg, MCV: 98fI, MCHC: 32.8g/dl, Platelet: 170 *103/μl, leu: 35, SGOT: 123 U/L[increased], SGPT: 118 U/L[increased]. The results of viral serology previously send to clinical laboratory were summarized in Table II.

folic acid (>20) [increased due to previous folate therapy], Vitamin B12: 112pg/ml (low), on bone marrow aspiration there had been an erythroid hyperplasia and megaloblastic changes noticeable and patient had been already taking folic acid and vitamin B12 (IM) to resolve the ineffective erythropoisis and megaloblastic changes and help to regenerate red blood cell production. Azathioprine (1mg/kg/day) was also added to the therapeutic regimen as the next line of immunosuppressive therapy to control the hemolysis.

According to above lab data, a high CMV IgM was detected and the first diagnostic impression was AIHA secondary to primary CMV infection, consequently CMV viral load was evaluated by real-time PCR (Taqman Technology) and the results were 25000

cpn/ml.

On consultation with hospital's infectious disease ward, Ganciclovir(5mg/kg/bd) was started for the patient and with another scheduled blood transfusion , he had been discharged from hospital with stable condition on August 2010. A consultation with Tehran university Children medical center, immunology allergy Research institute, due to fluctuation in CMV viral load, ongoing hemolysis and unresponsiveness to therapy had been ordered and the results were as follows:

IgM: 689mg/dL(NR,34-426mg/dL), IgG: 1046mg/dL(NR,217-904mg/dL), IgA:

43mg/dL(NR,11-90mg/dL), IgE: 4.5, CH50: 94%(NR,>90).and the result of flowcytometry evaluation were as follows:

CD3: 56% (NR, 55-82%), CD4: 8 %( NR, 27-59), CD8: 46 %( NR, 14-34%), CD19:14 % (NR, 9-22%, CD4/CD8) 0. 17 %( NR, 0.98-3.2%). According to this result patient has decreased CD4/CD8 ratio.

On August 2010, Patient therapy had been changed from ganciclovir to oral valganciclovir capsule (40mg/day) as a result of venous fragility and multiple ruptures due to venipuncture, but after 14 day red blood cell hemolysis accelerated and the drug had been reversed to ganciclovir Again there had also been a consultation with ophthalmologist for patient’s bilateral chorioretintis and the most possible etiology considered by the ophthalmologist was CMV chorioretintis secondary to CMV infection and it was recommended that differential diagnosis with Toxoplasmosis, HIV and EBV infection has to be considered. It had also been recommended by the ophthalmologist that ganciclovir be continued.

On January 2011 there had been a consultation with Shariati Bone marrow (BM) transplantation center for B.M transplant on the context of Immune deficiency or autoimmunity but due to his CMV infection he has not been considered as a candidate for bone marrow transplantation. On January, 2, 2011 as a result of uncontrolled hemolysis, unresponsiveness to therapy, high dependency on blood transfusion and increasing CMV viral load and upon consultation with infection disease ward it had been suggested by corresponding attendant that if CMV PCR is still positive, the resistance to ganciclovir should be investigated and foscarnate should be added to the regimen. At that time foscanate (80 mg/day) had been started but 3 days later on January, 5, 2011 due to impaired hepatic function and abnormal PT, PTT, he had been admitted to ICU with generalized icter, direct hyperbilirubinemia(total bilirubin 47mg/dl, direct bilirubin 30 mg/dl), hemoglobin 4.5 g/dl and direct coombs (3+ pos). HDMP hadbeen started again to control the hemolytic episode, Foscarnate had been discontinued and ganciclovir had been put on hold because of impaired liver function till January, 6, 2011 andfrom that date till almost his last days had been receiving ganciclovir nonstop. Due to unresponsiveness of patient to immunosuppressive and immunomodulatory drugs on January, 18, 2011 it was decided by hematology oncology ward responsible attendant to start a cycle of rituximab (100mg/4hr/wk for 4 wk) in order to control the hemolysis. There had been a good response, even after the first doses hemolysis decreased and hemoglobin started to rise and on January, 29, 2011 he was discharged from hospital and continued on rituximabonce a week and Ganciclovir, Vitamin K, E and ursodoil (150mg/day). On April 2011 there had been a CMV PCR of 9025 cpn/ml but following septicemia with Staph Aureus which he received vancomycin for it. His hemoglobin decreased, and upon CMV viral load quantization on may 2011 CMV viral load increased up to 3,084,354 cpn/ml , chorioretininot is flared up and patient's huge splenomegaly accelerated the RBC destruction cycle and thrombocytopenia. There had also been a hepatomegaly (span 10 cm), and an obvious facial bone deformity (chipmunk face) due to chronic hemolytic anemia and extramedullary erythropoisis. 

The results of patient's CBC parameters at that time were as follow:

WBC: 42.1 103/μl, RBC: 1.49 mil/ul, Hemoglobin: 5.2 g/dl, Hematocrit: 14.2%, MCV: 95fI, MCH: 34.9pg, MCHC: 36.6g/dl, Platelet<10000 /μl, Retic: 0.7%, RDW: 34%, 800 NRBC/ 100 WBC, macrocyte: (2 +), microspherocyte: (1+), schistosyte: slight, RBCagglutination: (2 +), megaloblastic and dyserytropoetic changes, cabot ring and also multiple Howell jolly bodies, giant band and hypersegmented neutrophiles was been observed. For better management of CMV infection it had been decided to start giving foscarnate to patient and fortunately on this occasion there had been a good response and there had been no hepatic toxicity and CMV PCR declined to 90251 cpn/ml and CMV Ag (PP65) became negative. Hemolysis was controlled and he was discharged from hospital with stable condition but again 2 weeks later following a diarrheal episode he had been admitted to the hospital with hemoglobin 6.5g/dl and he had a blood transfusion. He was discharged from hospital on stable condition and blood samples had been taken. On July 2011 for more evaluation of patient immunologic status and another flow cytometric evaluation which revealed almost the same results as the last time with decreased CD4/CD8ratio and increased CD16+56 31.63% (NR,5-15%) . On August 2011 at last admission he had fever and a WBC count of 14.2/ul ,Hemoglobin 4,2g/dl ,plt<10000/ul, Neut 54,lymph 3o%, Band 12%, Pro 2%, Myeo 3%, NRBC 30/100 WBC, Retic 7, 4, coombs 3+ positive, PT 17sec (increased) PTT (49 sec increased). Because the presence of fragmented RBCs in peripheral smear DIC was suspected. A positive blood culture with staph aureus was observed. His clinical course worsened and he had bloody endotracheal secretions as a consequence of pulmonary hemorrhage with rapid progression of cardiopulmonary failure and finally a cardiac arrest. Attempted CPR failed and ultimately he died on September 2011 on 6 pm.

**Table I:** TORCH study of patient’s mother

	**Toxo IgG **	**Toxo IgM **	**Rubella IgM**	**Rubella IgG**	**CMV IgM**	**CMV IgG**	**HSV Ab1+2 IgG**	**HSV Ab1+2 IgM **
	<0.1	0.2	0.4	93.6	0.3	1.4	14.0	0.3
**Neg or NR**	< 1	< 0.8	<0.8	< 10	0.3	>0.6*	< 16	<0.9

Toxo: Toxoplasma, CMV: Cytomegalovirus, HSV: Herpes Virus, Neg: Negative, NR : Non reactive

* Immune

**Table II:** Viral serology of patient with CMV

**Test**	**EBV IgG (U/ml)**	**EBV IgM (U/ml)**	**HAV Ab total ** **(Iu/L)**	**HAV IgM (U/ml)**	**CMV IgM** **(IU/ml)**	**CMV IgG** **(IU/ml)**
Result	68	0.4	60	93.6	5.2	6.3
Test	HBC Ab	HCV Ab	HIV Ab	HBS Ag	HBS Ab ( Iu/L)	
Result	0.5	0.1	0.3	0.5	297	

## Discussion

Here we report a child with a history of transfusion dependent refractory AIHA secondary to primary CMV infection and almost unresponsive to therapy.

When a patient presents with a history and clinical signs that are consistent with AIHA, multiple clinico-pathological parameters must be considered in the diagnosis of this disease.

These include the presence of anemia, auto agglutination, spherocytes positive direct anti globulin (Coombs’) test, and the elimination of any other underlying causes of anemia. No single finding is pathogonomic for AIHA, so careful interpretation is important. The anemia of AIHA is often severe, with a PCV of less than 20%. The anemia is usually regenerative, since erythropoeisis is not adversely affected unless the immune responseaffects hematopoietic cells which in our case this could be the cause of anemia despite of receiving folate and B12. One-third of all cases of AIHA present with a non regenerative anemia, possibly due to acute disease onset and lack of time for a regenerative response.

Autoimmune hemolytic anemia in children may be associated with immunodeficiency syndrome, malignancy, and multisystem autoimmune disorders. The pathogenesis of anti erythrocyte autoantibody formation in AIHA is unclear but may involve an impaired immunoregulatory mechanism. Immunosuppressive therapy with corticosteroids is the first line of therapy in warm type AIHA and a response has been observed in approximately 80%of cases. Corticosteroids are believed to inhibit Fc-receptor mediated clearance of IgG sensitized erythrocytes in the spleen, and may inhibit autoantibody synthesis. IVIG has a role in patients with AIHA who do not respond completely to conventional dose of steroids. IVIG is able to produce a potent blockage of the reticuloendothelial system; thereby inhibiting phagocytosis of IgG sensitized red cells. Our patient did not respond completely to conventional dose of steroids, and for this reason we initiated HDMP and IVIG. He initially showed good responsebut hemolysis continued. 

Cases not remitting with this strategy may benefit from splenectomyor other immunomodulatory drugs. Splenectomy is an alternative therapeutic option forpatients with AIHA who require high maintenance prednisolone doses or who have multiple and frequent relapses. Splenectomy removes a major portion of both the phagocytic reticuloendothelial system and the autoantibody-producing B cells. Splenectomy is sometimes considered for children with chronic AIHA, although it should be avoided in young infants because of the high risk of sepsis and mortality. In our case, because of the age he was not considered as a candidate for this procedure but it had been considered as an option in the future if the remission would not be achieved. Azathioprine which works by decreasing the production of white blood cells by interfering with the production of the cell’s genetic material, and DNA, stops the cells from dividing and multiplying, which in our case had been the next option to control hemolysis but as a result of reoccurrence of hemolytic episodes after any kind of infection and dependency on blood transfusion despite of combination multidrug treatment there had been no choice except to use a next more potent immunomodulatory drug. Rituximab or the anti-CD20 antibody which is a chimeric, human, IgG1/κ monoclonal antibody (MoAb) specific for the CD20 antigen, expressed on the surface of B lymphocytes.

This antibody has induced rapid in vivo depletion of both normal B lymphocytes and lymphoma B cells (10). This drug has been used in therapy-refractory AIHA, mostly in children and in a small number (<30) of adults (2-4). The outcome is unpredictable, varying from ineffective to a high response rate (10). 

In our case, there had been a good response even after the first dose and there had been a rise in hemoglobin. A few days later an infection episode hemolysis accelerated and CMV count rose up. In order to contain the CMV infection foscarnate had been added to the therapeutic regimen and there had been a good containment of CMV infection and negative CMV - Ag ever since but evaluation of immunologic workup had been ordered and probable candidacy for bone marrow transplantation on the context of immunodeficiency or autoimmunity had been still on the table. Chorioretinitis in our case had been another complication of CMV infection, and inflammation which is usually caused by congenital viral, bacterial, or protozoal infections in neonates. Congenital toxoplasmosis and cytomegalovirus (CMV) infection are the most common etiologies in this age group. Patient’s immunologic status showed a decreased CD4/CD4 ratio which is pathognomonic for HIV infection but since his HIV antibody was negative in two occasions, HIV infection was ruled out. The increase in CD8 positive cells is not unique to HIV-1 infection, since many viruses and vaccinations cause a transient increase in the CD8 population. Generally, the CD4/CD8 ratio is increased in autoimmune diseases and decreased in viral infections. Cyclosporine and prednisone therapies decrease theCD4/CD8 ratio. In our case prednisolone could be considered responsible for this decline inCD4/CD8 ratio. Disseminated intravascular coagulation (DIC) is a manifestation of an underlying pathologic process such as cancer, infection, trauma, or obstetric catastrophe.

It can manifest as thrombosis, bleeding, or both. DIC is due to excessive activation of coagulation. Unregulated activation of the haemostatic system results in the clinical syndrome known as DIC. Excessive activation of coagulation, coupled with the inability to neutralize circulating activated procoagulants as physiologic inhibitors are overwhelmed, distinguishes DIC from physiologic clotting.

Potent thrombogenic stimuli cause uncontrolled, infection is a common cause of acute, severeDIC.4 which can be caused by nearly any type of microorganism. Components of these microorganisms activate cytokines (chiefly tumor necrosis factor and interleukin-6), inducing an inflammatory response and triggering coagulation. Anything that enhances the spread of the infection 

(immunosuppression, hepatic insufficiency, or functional or anatomical asplenia5) can foster the development of DIC.

## Conclusion

Clinicians should consider CMV infection in the differential diagnosis of hemolytic anemias.

The true incidence of this complication may be underestimated, because CMV serology may not be routinely obtained in patients with hemolysis. Possible therapeutic options include antiviral therapy and steroids, although the best treatment strategy is still controversial. Hematopoietic stem cell transplantation has also been reported in occasional patients (7). However the side effect of prolonged administration of immunosuppressive drugs or stem cell transplantation is substantial (8). Splenectomy which was one of our options in order to decrease transfusion dependency but we never had the chance to perform it since on the last admission his conditions worsened and he died with severe anemia, septicemia, pulmonary hemorrhage as a consequence of DIC, and cardiovascular failure.

## Conflict of interest

The authors have no conflict of interest.
